# Radiofrequency Ablation of Uterine Fibroids: a Review

**DOI:** 10.1007/s13669-016-0183-x

**Published:** 2016-11-04

**Authors:** Bruce B. Lee, Steve P. Yu

**Affiliations:** 1 Santa Monica-UCLA Medical Center, 1250 16th Street, Santa Monica, CA 90404 USA; 2 Roxbury Clinic and Surgery Center, 465 N Roxbury Dr. #1001, Beverly Hills, CA 90210 USA; 3 Ventura Surgery Center, 1752 S Victoria Ave #A, Ventura, CA 93003 USA; 4 Division of Minimally Invasive Surgery, Department of OBGYN, University of California|, Los Angeles, CA USA

**Keywords:** Uterine fibroids, Radiofrequency ablation, Minimally invasive surgery, Laparoscopy

## Abstract

Laparoscopic, ultrasound-guided radiofrequency ablation (RFA) is a new, FDA-cleared uterine sparing, outpatient procedure for uterine fibroids. The procedure utilizes recent technological advancements in instrumentation and imaging, allowing surgeons to treat numerous fibroids of varying size and location in a minimally invasive fashion. Early and mid-term data from multi-center clinical trials have demonstrated safety and efficacy, with resolution or improvement of symptoms and significant volume reduction. Re-intervention rates for fibroid symptoms have been low. The procedure is well tolerated with a typically uneventful and rapid recovery requiring NSAIDs only for postoperative pain. While post RFA pregnancy data are limited, the results are promising.

## Introduction

Uterine fibroids remain a major women’s health issue with significant economic and reproductive impact. They occur in almost 70 % of Caucasian women and in greater than 80 % of African American women by age 50 [1]. Fibroids have long been known to be the most common indication for hysterectomy. In an analysis of new diagnoses of fibroids in U.S. women with access to medical care, Cardozo estimated the total direct and indirect 2010 cost of uterine fibroids (including associated obstetrical complications) to be $5.89–$34.37 billion annually [2].

While 30 % or 12.3 million U.S. women between the ages of 25 and 44 experience symptoms of fibroids [3], the number of hysterectomies, myomectomies, and uterine artery embolization procedures total less than 250,000 annually [4]. This large discrepancy may be due to factors such as culture, limited access to medical care, cost factors, and work-related issues. More likely, the data indicates patient rejection of the procedures offered. Radiofrequency ablation of fibroids was developed to provide women with a new minimally invasive option for fibroid treatment that is safe, effective, and uterine conserving.

## RFA Historical Events

The basic technique of RFA was first described by D’Arsonval in 1891 [5]. He showed that passage of a RF current through tissue caused an increase in tissue temperature.

In 1910, Beer reported the treatment of bladder neoplasms through a “cauterizing cystoscope” [6]. In 1911, Clark described treatment of superficial malignant growths with oscillatory desiccation [7]. RF did not become widely used in medicine until Cushing and Bovie introduced the Bovie knife [8] in 1928. Radiofrequency catheter ablation [9] was developed in the 1980s and initially utilized a direct current (DC) between a catheter electrode and a cutaneous surface electrode to treat tachyarrhythmias. Radiofrequency ablation subsequently supplanted DC ablation and is now widely adopted and first line therapy for certain tachycardias.

**Fig. 1:**
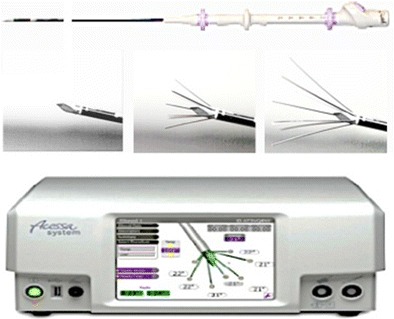
Acessa System consisting of deployable multi-electrode array and RFA generator

Tumor destruction using RFA is a recent innovation. In 1990, McGahan et al. [10] and Rossi et al. [11] independently reported production of coagulative necrosis using a proximally insulated electrode with an exposed tip. The insulation of the proximal portion of the electrode allowed insertion deep into tissue to produce a distal ablation at the tip. In 1993, McGahan [12] utilized this technique to treat liver tumors in humans. However, practical application of RFA was limited by the diminutive ablation size and the elongated, oval shape characteristic of a single electrode device. Various device modifications ensued, including multi-electrode arrays, cluster electrodes, internally cooled electrodes, pulsed RF application, saline infusion during RFA, and techniques such as pharmaceutical therapy and vascular occlusion to decrease heat loss. [13–16]. Ablation diameters approaching 7 cm are now achievable in some cases. Such advancements led to successful treatment of osteomas and subsequent FDA clearance of two systems, one in 2000 for the specific treatment of non-resectable liver lesions and another in 2004 for non-resectable liver tumors. The FDA has also cleared several systems for metastatic bone lesions. In 2012, the FDA cleared the Acessa System (Halt Medical, Inc., Brentwood, CA) (Fig. 1) for “ablation of soft tissue including treatment of symptomatic uterine fibroids under direct ultrasound guidance.” Radiofrequency tumor ablation has been used with varying success in a number of other sites including the lung, kidney, adrenal, thyroid, breast, bone, thoracic neoplasms, and uterus (endometrium, fibroid tumors) [17–23].

## Principles of Radiofrequency Ablation

Radiofrequency ablation (RFA) is a form of hyperthermic ablation, i.e., using elevated temperature to produce tissue destruction. Heat may be applied by direct thermal conduction as in endometrial ablation with heated fluid or by ultrasound or electromagnetic (RF, laser, microwave) energy. RFA is the specific process of applying energy in the form of an alternating current in the radio frequency range (between 3 kHz and 300 GHz). Medical procedures typically use frequencies between 450 to 500 kHz. Nerve stimulation does not occur during RFA since stimulation of nerves occurs at frequencies below 10 kHz [24]. RFA is generally predictable, reliable, and effective, given an appropriate target tissue.

Broadly, a radiofrequency system consists of a generator, an electrode, electrode return pads, and cables connecting these elements. Typically, RF systems use either temperature or impedance feedback control and are monopolar.

The generator produces a high frequency, low voltage, alternating current that is transmitted via an electrode with an insulated shaft. Placement of the electrode into a target tissue results in transmission of the current through the tissue with a particular point of entry. The current then travels to the electrode return pads and back to the generator, completing the circuit. Heat is produced by ionic (Na, K, Cl) movement (resistive heating) within the cells adjacent to the exposed portion of the electrode in response to the very rapid shifts in polarity inherent with an alternating current. The heat produced then spreads by simple thermal conduction, producing a volumetric ablation through coagulative necrosis. The size and shape of the ablation volume is determined by the temperature reached, the length of time at that temperature, and the shape of the electrode (i.e., single electrode, multiple electrodes in an array, curved or straight electrodes). Additional factors influencing ablations include tissue conductivity and the presence or absence of fluid collections or vascularity which act as “heat sinks.”

Human cells die nearly immediately at 60 °C. Achievement of cell death requires longer exposure at lower temperatures [25]. High temperatures are desirable to produce a larger ablation volume in a shorter amount of time. However, temperatures greater than 100 °C produce local tissue charring and vaporization which cause lower current density and insulation, decreasing both heat generation and conduction. This results in potential cold areas and incomplete ablations.

Significantly, the interface between the return electrodes and the body is another site of heat generation. As a result, prolonged application of RF energy may potentially result in skin burns near the leading edge of the return pads. Pads with larger surface areas dissipate heat better and minimize the temperature rise. Some RF systems include thermocouples in the return pads to monitor skin temperature as a safety feature.

## Development of RFA of Fibroids

The first reported application of RFA to treat uterine fibroids was by Lee [26] in 2002. This initial report described treatment from 1999 to 2001 of 52 patients with 197 myomas ranging in size from 1 to 11 cm. He reported uterine volume reductions of 36 and 41 % at 3 and 6 months, 12-month symptom resolution rates of better than 90 %, and no intra- or perioperative complications, readmissions, or re-interventions. In 2005, Lee [27] reported the 3-year outcomes. Ninety-four percent of patients were asymptomatic, with one cumulative re-intervention for fibroid symptoms at 23 months. Lee also launched a prospective trial in 2002 utilizing a radiofrequency ablation needle shaped in a starburst pattern (Starburst XL, Rita Medical) and incorporated preoperative and postoperative evaluations using the Uterine Fibroid Symptom and Health-Related Quality of Life questionnaire developed by Spies [28]. In 2005, Lee initiated design of a new radiofrequency ablation system specifically to treat uterine fibroids. This system, currently called the Acessa System, ultimately received FDA clearance in 2012 for the treatment of uterine fibroids.

In 2005, Bergamini et al. [29] reported a pilot study of RFA of fibroids in 18 subjects without intraoperative ultrasound guidance using the Rita Medical RFA system and Starburst XL needle. They reported reductions in myoma volumes of 41.5, 59, and 77 % at 1, 3, and 6 months with significant improvement in the symptom and quality of life scores at 3 and 6 months.

In 2007, Milic et al. [30] reported laparoscopic ultrasound-guided RFA using the LaVeen Needle Electrode (Boston Scientific, Natick, MA) in four patients with small, symptomatic fibroids. The procedure was not technically feasible in one patient, and only one of the remaining three had symptomatic relief at 7 months.

In the same year, Lee [31] reported 36-month data from a prospective trial of 110 patients. He also reported the first known pregnancy and delivery after RFA of fibroids. At 3, 12, and 36 months, uterine volume declined by 39, 50, and 46 %, respectively. UFS-QOL symptom scores fell 67, 87, and 90 %.

In 2011, Garza et al. [32] published results of a feasibility study using the Acessa procedure. Seventy-six myomas in 31 subjects were treated with statistically significant reductions in uterine volume and UFS-QOL questionnaire scores. There were no repeat treatments, repeat hospitalizations, or device-related complications. These results were confirmed by Robles et al. [33] in 2013.

In 2013, Chudnoff et al. [34•] published the pivotal, multi-center clinical trial results that lead to FDA clearance of the Acessa System in 2012. The study was a prospective multi-center international trial of 137 subjects with documented fibroids and menstrual blood loss between 150 and 500 mL. Baseline MRI was used to establish fibroid number and size and to exclude adenomyosis and type 0 submucosal myomas. The three primary end points were volume of menstrual bleeding at 12 months compared to baseline, surgical re-intervention rate for heavy menstrual bleeding at 12 months, and device and procedure-related adverse events within 12 months. Secondary endpoints were uterine volume measurements, UFS-QOL scores, overall treatment evaluation scores, and general health outcome scores at 12 months. Menstrual blood loss fell in 81.9 % of subjects with a decrease of at least 50 % in 40.2% of subjects and 22 % in 67.7 %. There was one surgical re-intervention for persistent bleeding and one serious adverse event. Total mean myoma volume decreased 45.1 % from baseline. Ninety-four percent of subjects were satisfied with their treatment.

In 2014, Berman et al. [35•] reported the three-year findings of the same clinical trial. The cumulative re-intervention rate for fibroid symptoms was 11 %. Seven of the fourteen patients who had another procedure were found to have adenomyosis. All subscale UFS-QOL scores and all health state scores remained stable compared to one-year results indicating durability.

Brucker et al. [36•] reported in 2014 the result of a randomized, prospective clinical trial evaluating RFA of fibroids with the Acessa System and laparoscopic myomectomy. Twenty-five patients were treated in each group. Operative blood loss and hospital stay were both statistically significantly lower in the RFA group. Ninety-eight percent of fibroids visualized intra-operatively by laparoscopic ultrasound were treated in the RFA group versus 80.3 % in the myomectomy group. The operative time was also lower in the RFA group.

A randomized clinical trial comparing RFA of fibroids, laparoscopic myomectomy, and uterine artery embolization (TRUST study) [37] is currently underway using the Acessa System.

Garza-Leal [38] reported a feasibility and safety study of 19 subjects and 20 fibroids in 2011 using a combined ultrasound and radiofrequency device (VizAblate, Gynesonics, Redwood City, CA) via an intrauterine approach. Brölmann et al. [39] in 2016 reported 12-month results with the same device in 50 patients with 118 target fibroids (mean 2.4 fibroids/patient), fibroids with a mean fibroid diameter of 2.9 cm (range 1.0 to 6.9 cm). Ablations were achieved in 92 (78 %) of 118 target fibroids with an average reduction in perfused volume of 68.1 and 67.4% at 3 and 12 months, respectively. Sixty-four percent (59/92) of fibroids treated were Type 1 or 2. Symptom severity scores decreased 55.1 % and menstrual pictogram scores decreased 53.8 % by 12 months. Four patients (8 %) underwent re-intervention by 12 months, and there were 34 adverse events with two readmissions within 30 days of the procedure. Satisfaction rate was 87.8 %. A pivotal multi-center clinical trial was initiated in 2014.

In 2015, a case report of intrauterine adhesion formation after trans-vaginal, RFA of fibroids was published [40].

International interest in RFA of fibroids has extended to Denmark [41], Korea [42, 43], China [44, 45], and Italy [46]. Results of a trans-vaginal, extra-uterine approach with trans-vaginal ultrasound have also been reported [42].

## The Acessa Procedure Using the Acessa System

Physician qualifications:

1. Physicians should be experienced laparoscopic surgeons and must be competent in the performance of and interpretation of pelvic ultrasound.

Equipment requirements:

1. Standard laparoscopic tower (insufflator, camera box, light source, printer)
2. Laparoscope 5 or 10 mm, zero degree
3. RFA generator, foot pedal, electrode dispersive pads, cables
4. RFA handpiece (electrode)
5. Ultrasound machine with laparoscopic transducer (side firing, variable frequency, either linear or sector, rigid)
6. Two video monitors placed adjacent to one another, one for the laparoscopic image and one for the ultrasound image.

RFA of fibroids is a laparoscopic procedure utilizing a 5-mm port for the laparoscope and a second 10–12-mm port for a laparoscopic, rigid, side-firing ultrasound transducer. The author prefers patients to be in the dorsal position with a single-tooth tenaculum applied to the cervix to manipulate and stabilize the uterus. The RFA handpiece is inserted percutaneously through a 2-mm skin incision and directed into each myoma with laparoscopic and ultrasound guidance. Ultrasound is utilized to verify appropriate placement of the device within each myoma. The deployable electrode array is generally used for ablations exceeding 2 cm and is retracted at the completion of the ablation. Coagulation of the track is then performed with withdrawal of the handpiece. Irregular myomas and those with diameters 4 cm and greater often require multiple overlapping ablations in order to ensure adequate ablation of the myoma periphery.

After ablation, myomas are not replaced by fibrous tissue but gradually reabsorbed by the surrounding myometrium. Complete myoma reabsorption is common with medium- to small-sized myomas. The likelihood of complete reabsorption is dependent upon the completeness of ablation, time, and the myoma location.

## Pregnancy Data

As of the date of this writing, documentation exists of 15 pregnancies in 13 patients yielding 13 live births after RFA of fibroids. Fourteen pregnancies occurred in 12 patients after laparoscopic, ultrasound-guided RFA and one pregnancy occurred in a patient who underwent trans-uterine RFA. In the laparoscopic group there were 12 viable, term infants and 2 first trimester spontaneous abortions. The age of two women who underwent spontaneous abortion was 38 and 41 years at treatment. The overall average age of the 14 women who had laparoscopic RFA of fibroids was 35.7 yrs. The average interval between the RFA of fibroids and first postoperative conception was 7.7 months. Two women had two pregnancies each post RFA. Of the 13 births, 8 were cesarean deliveries and 5 were vaginal deliveries.

Additional pregnancy data will no doubt become available over time. Since RFA of fibroids causes minimal myometrial damage, RFA clinical trials such as the University of California Ultra Study and the ongoing TRUST randomized clinical trial allow inclusion of subjects who wish to conceive after treatment. A German clinical trial is also underway which is evaluating obstetrical outcome after RFA of fibroids as its primary endpoint.

## Clarifying Misconceptions About RFA of Fibroids

New procedures, being new, are inherently susceptible to misunderstanding and misinterpretation because published data from rigorous clinical studies remain largely unknown for a period of time. The following myths are at times encountered by the author and merit attention.

1. RFA of fibroids is the same procedure as myolysis.

The term myolysis may be broadly interpreted as a procedure that destroys muscular tissue. In this regard, RFA of fibroids could be called a form of myolysis. However, the former procedure known as myolysis was very different in how it was performed and in its effects upon the uterus and fibroids.

Donnez et al. [47] reported using a Nd:YAG laser placed hysteroscopically to treat submucosal myomas (Donnez). Goldfarb [48] reported the first U.S. series using the Nd:YAG laser laparoscopically and subsequently reported using bipolar needles to repeatedly pierce and coagulate myomas and their peripheral vessels [49]. Unfortunately, these techniques caused serosal and myometrial damage, resulting in adhesion formation, subsequent uterine rupture in pregnancy, and fetal loss [50, 51]. Elective cesarean section was recommended for those pregnancies that reached term.

In contrast, RFA of fibroids was designed to volumetrically ablate fibroids with very minimal damage to myometrium and serosa. The insulated shaft allows treatment of fibroids deep within the myometrium without ablation of proximal myometrium. Electrode insertion does result in a needle track several millimeters in width and a serosal puncture. If excessive coagulation of the needle track is avoided, myometrial and serosal damage should be minimal.

For this reason, in pregnancies after RFA, vaginal deliveries have been permitted by some providers. Moreover, in the author’s experience, in cases where RFA of fibroids was the sole procedure performed, evaluation at subsequent procedures do not reveal pelvic or abdominal adhesions. Similarly, in the limited number of pregnancies after RFA, serial ultrasound evaluations during pregnancy and palpation and inspection at cesarean section have not revealed the presence of myometrial thinning or “uterine windows” at ablation sites. Nonetheless, at this time, the data regarding post RFA pregnancies is still very limited and a larger clinical experience is needed before definitive recommendations can be made.

2. Only fibroids that are submucosal cause heavy menstrual bleeding.

In the pivotal phase three multi-center trial reported by Chudoff et al. in 2013, all study subjects had uterine fibroids and a baseline menstrual blood loss between 160 and 500 mL based on hemoglobin measurement from catamenial products by the alkaline hematin method. In a retrospective analysis of this prospective trial, Galen et al. [52•] reported that only 48.4 % of subjects (59 of 122 who submitted catamenial products at 12 months) had one or more submucosal myomas. Moreover, in the 51.6 % of subjects without submucosal fibroids, RFA of fibroids decreased menstrual bleeding 31.8 % compared to baseline. While additional studies are needed, this evidence strongly suggests that intramural myomas contribute to heavy menstrual bleeding and that heavy menstrual bleeding in patients with fibroids does in fact occur in the absence of submucosal leiomyomas.

3. With RFA of fibroids, the “problem is the recurrence rate.”

In 2014, Berman et al. reported data on 104 subjects who had 36-month data post RFA of fibroids. The cumulative re-intervention rate was 11 %. These data compare favorably with that of laparoscopic myomectomy and uterine artery embolization.

Within the group of subjects who had additional procedures because of excessive menstrual bleeding, pathology, or additional magnetic resonance imaging studies identified adenomyosis in seven subjects. If these subjects had been excluded (suspected adenomyosis by baseline MRI was an exclusion criteria in the study), the re-intervention rate would have actually been 5 %.

In addition, patient reported data (Uterine Fibroid Symptom and Health-Related Quality of Life questionnaire) were durable, demonstrating stability of the significant improvement in symptom scores compared to baseline at 12, 24, and 36 months.

4. Disadvantages of the Halt System include additional percutaneous skin incisions, its treatment of one fibroid at a time (<8 cm diameter), and its ablation of fibroids centrally while fibroids grow peripherally.

The skin incisions required when performing RFA of fibroids is one 5-mm incision for a 5-mm laparoscope, a 10–12-mm incision for the laparoscopic ultrasound transducer, and then a variable number of 2-mm incisions for percutaneous placement of the electrode handpiece. The novice will often require 3 to 4, 2 mm incisions whereas with greater experience, these may be limited to one or two. The incision requirement (two trocar sites) is approximately 50 % of traditional laparoscopic hysterectomy or laparoscopic myomectomy.

The clinical investigators participating in the multi-center, pivotal phase three clinical trial reported by Chudnoff et al. had very limited or no prior experience with RFA of fibroids. Therefore, practical considerations and concern for patient welfare demanded limitations on the number, size, and overall volume of fibroids. The existence of these limitations is not an indication that uteri with fibroids of greater size or number cannot or should not be treated. Indeed, the experience and ability of the surgeon is a limiting factor in all procedures. Using RFA, the author commonly treats fibroids 10–15 cm in diameter and frequently treats uteri that are 20–26 weeks in size.

As a general rule, fibroids of 1.5 cm in diameter do not require deployment of the electrode array to ablate the myoma periphery. Fibroids between 2 and 3.5 cm that are roughly spherical should be treated by inserting the tip of the RFA handpiece in the midline of the fibroid and directed toward the center. Appropriate deployment of the electrode array will result in treatment of the myoma periphery. In fibroids ≥4 cm in diameter, multiple eccentric ablations are generally required with the goal of treating the periphery of the myoma, not the center.

5. RFA can treat only a limited number of fibroids in certain locations.

As in all procedures, greater experience and instruction increases skill level and ability. Ideally, initial cases should be limited to those with one to three medium-sized fundal myomas. The author currently averages approximately 15 myomas per case (range 1 to 45). With experience and additional training, greater numbers in more challenging locations may be treated. In terms of location, one of the advantages of RFA of fibroids is the eventual ability to treat myomas located in sites traditionally regarded as high-risk with safety and relative ease. Such myomas include cervical, broad ligament, and round ligament myomas.

6. RFA damages the endometrium.

The basic premise of RFA of fibroids is to treat as much of each myoma as possible and to spare non-fibroid tissue. The procedure and instrumentation has been designed to allow surgeons to accomplish this objective. With adherence to the standard protocol for treating myomas, damage to endometrial tissue should be minimal or absent. For example, posterior myomas are treated by entering the uterus through the serosa of the posterior uterine wall. This laparoscopic approach allows direct access to the myoma avoiding entering or passing through the endometrial cavity. In contrast, trans-cervical hysteroscopic approaches using mechanical dissection or radiofrequency ablation necessitate cervical dilatation and at least some degree of endometrial damage.

Experienced surgeons commonly treat type 1 and type 2 [53] submucosal myomas (greater than 50 % and less than 50 % intracavitary, respectively). It is recommended that type 0 myomas (100 % intracavitary) be removed via hysteroscopic myomectomy.

## Conclusions

RFA of fibroids has proven to be safe, versatile, and effective in reducing or eliminating symptoms related to uterine fibroids. It is effective in treating fibroids of various sizes and number in a single setting. Recovery is rapid and usually uneventful with mild postoperative pain. Return to work frequently occurs by postoperative day four. Moreover, the potential for RFA of fibroids to produce health care savings is significant. Rapid return to work, a low re-intervention rate, and perhaps a lower elective cesarean rate compared to myomectomy are positive factors in this regard. Greater experience with pregnancy outcomes after RFA of fibroids will provide valuable data and will ultimately influence the adoption of this minimally invasive treatment. Finally, as with all new procedures, a proper understanding of the procedure, adequate training, and knowledge of one’s surgical abilities are essential to maximizing patient outcomes.
